# Participation as a Predictor of Quality of Life among Japanese Children with Neurodevelopmental Disorders Analyzed Using a Machine Learning Algorithm

**DOI:** 10.3390/children11050603

**Published:** 2024-05-16

**Authors:** Hiroyasu Shiozu, Daisuke Kimura, Ryoichiro Iwanaga, Shigeki Kurasawa

**Affiliations:** 1 Department of Occupational Therapy, College of Life and Health Sciences, Chubu University, Kasugai 487-8501, Japan; 2 Department of Occupational Therapy, Faculty of Medical Science, Nagoya Woman’s University, Nagoya 467-8610, Japan; dkimura@nagoya-wu.ac.jp; 3 Department of Occupational Therapy Sciences, Graduate School of Biomedical Sciences, Nagasaki University, Nagasaki 852-8520, Japan; iwanagar@nagasaki-u.ac.jp; 4 Department of Occupational Therapy, School of Health Sciences, Fukushima Medical University, Fukushima 960-1295, Japan; kurasawa@fmu.ac.jp

**Keywords:** participation, well-being, QOL, neurodevelopmental disorder, children, random forest analysis

## Abstract

Participation is important for children’s quality of life (QOL). This study aimed to identify participation factors that influence QOL among Japanese children with neurodevelopmental disorders. Ninety-two Japanese parents of children with neurodevelopmental disorders participated in this study. The parents completed the parent version of the Kid- and Kiddo-KINDL health-related QOL questionnaire and the Participation and Environment Measure for Children and Youth. The data were examined using the random forest algorithm to analyze the participation factors that affected the children’s QOL. The analyses revealed that school and community environmental factors that affected participation were the most important predictors of QOL among children. As school and community environments can significantly impact the QOL of children with neurodevelopmental disorders, greater focus should be placed on participation in environmental contexts.

## 1. Introduction

The World Health Organization has defined participation as active “involvement in a life situation”, such as engaging in school/educational, work, religious, leisure, recreational, and social activities [1]. Participation allows children to acquire skills and competencies, connect with others and their communities, and find purpose and meaning in life [2]. However, children who are experiencing illness, injury, disability, or disadvantage are often excluded from personal or cultural life situations that can give value to them and their families [3]. Previous studies have found that children with a disability tend to experience greater participation restrictions than those without a disability [4,5,6].

However, there is a persistent gap between knowledge and practice regarding encouraging participation in children [3]. Although participation has been recognized as important to general health and well-being, assessment and intervention practices have focused on changing body structures and functions and developing activity competencies, neither of which necessarily encourage participation in everyday life [3,7]. To promote participation-based practices and bridge the gap between knowledge and practice, more research is needed on developing programs to encourage participation and well-being in children with disabilities. Therefore, in a previous study, we examined the participation strategies used by parents of children with neurodevelopmental disorders (NDDs) [8]. Using co-occurrence network analysis and correspondence analysis, we analyzed strategy text data from the Participation and Environmental Measure for Children and Youth (PEM-CY) [8]. The result of the co-occurrence network analysis showed that the commonality of strategies that enable participation in home, school, and community settings is that parents explain their children’s characteristics and make others understand them in each setting [8]. We considered the importance of strategies to improve attitudinal environments and promote the participation of children with NDDs. This strategy could reduce stigma, which was a challenge in the public sphere (school and community) in a previous study. Therefore, specific strategies are required for each setting, suggesting the importance of context-specific approaches [8]. However, the study did not guide us as to which environments would be the most important to address in order to improve participation. Therefore, quantitative analysis is necessary to derive specific directions for developing a context-specific approach.

A survey conducted by the Japanese Ministry of Education, Culture, Sports, Science, and Technology found that 8.8% of junior high school students with NDDs who needed special support were enrolled in regular classes [9]. As this is a significant increase from the percentage of students with NDDs (2.5%) enrolled in regular classes 10 years ago, enhancing school support has become more important. Therefore, clarifying the structural relationships between participation and well-being, such as quality of life (QOL), for Japanese children with NDDs is necessary to develop appropriate interventions. Evaluating the association between children’s participation and well-being is complex, because many contributing factors mediate this relationship [10]. Therefore, this study measured QOL using the parent version of the Kid- and Kiddo-KINDL (KINDL) and measured participation using the PEM-CY, after which the relationships were analyzed using random forest analysis. This study aimed to clarify the relationship between QOL and participation for Japanese children with NDDs, thereby considering the need for a participation-focused approach in the future.

## 2. Materials and Methods

### 2.1. Study Design

This cross-sectional study was conducted from April to June 2023 to investigate participation status as a predictor of QOL among Japanese elementary school children with NDDs. We had already analyzed textual data on participation strategies from the same dataset [8]. The results showed that different strategies were implemented to address the participation difficulties of children with NDDs. Therefore, our next step was to identify the relationship between the QOL and participation status of children with NDDs. To do so, we analyzed the same data using random forest analysis, which is a type of machine learning algorithm.

### 2.2. Participants

The criteria for participants in this study were based on our previous study [8]. We enlisted the participation of parents whose children with NDDs were receiving treatment at a pediatric rehabilitation hospital in Japan. The eligibility criteria included children who were elementary school students aged 6 to 12 years (from first to sixth grade) and had been diagnosed with an NDD based on the Diagnostic and Statistical Manual of Mental Disorders—5th Edition by either a pediatrician or a child psychiatrist. In Japan, there are four types of educational settings for elementary students: regular classes, special support services in resource rooms, special support classes, and special support schools. In this study, children with NDDs in any of the four educational settings were included as research subjects.

### 2.3. Measures

#### 2.3.1. Parent Version of the Kid- and Kiddo-KINDL (KINDL)

The KINDL was designed to measure a child’s QOL. This study used the version that assesses children from the parents’ perspective [11,12]. This version is developed for elementary and junior high school students, and the Japanese version is standardized for elementary school students (i.e., 6–12 years). The KINDL comprises six subscales and 24 items, each of which is responded to using a 5-point scale. The six subscales are Physical Well-Being (e.g., illness, pain, and fatigue), Emotional Well-Being (e.g., boredom, loneliness, and fear), Self-Esteem (e.g., pride and feeling on top of the world), Family (e.g., relationships with parents and home conflicts), Friends (e.g., getting along and feeling different from others), and Everyday Functioning at School (e.g., enjoying class and worrying about the future). In this study, each subscale score was transformed to a 0–100 range.

In a previous Japanese study of elementary school children without disabilities in grades 1–6, the Cronbach’s alpha for the total KINDL scale was 0.87 and ranged from 0.58 to 0.85 for the subscales [12]. Note that there have been cross-sectional studies in Japan using the PEM-CY on children with physical disabilities [13] and on children with autism spectrum disorder [14].

#### 2.3.2. Participation and Environment Measure for Children and Youth (PEM-CY)

The PEM-CY is an evaluation tool reported by parents that gauges the involvement of children and youth, both with and without disabilities, and the environmental influences that impact their engagement in three distinct environments: home, school, and the community [15,16,17]. The measure has two sections: a participation section and an environment section. The participation section measures three participation dimensions in each setting: frequency (8-point scale from never (0) to daily (7)), extent of involvement (5-point scale from minimally involved (1) to very involved (5)), and desire for change (yes or no; those who answer “Yes” then select the type of change desired from a list of 5 options (e.g., more involved, less involved)). The participation section of the PEM-CY has 25 items assessing a child’s participation in activities at home (10 items), at school (5 items), and in the community (10 items).

The environment section measures the environmental support and barriers that influence the children’s participation in each setting: home (12 items), school (17 items), and community (16 items). This study used the overall environmental support score, which was the sum of all section scores, and converted it into percentages.

In addition, for each setting, parents could enter up to three strategies for encouraging their child’s participation. However, this study did not analyze textual data on the participation strategies of children with NDDs reported in the PEM-CY.

In a scoping review [18], the internal consistency of the PEM-CY was found to be moderate to good (0.59 and above) across the different scales. The measure also demonstrated good test–retest reliability for periods of one to four weeks (0.58 and above).

### 2.4. Procedure

Parents received both a written and verbal description of this study from their child’s therapist. They were also assured that their decision to participate or not participate in the study would not influence any rehabilitation interventions. If they agreed to participate in this study, they completed the KINDL and PEM-CY. The KINDL and PEM-CY were completed by parents who were familiar with their children’s situation. When completing the questionnaire, the therapist first explained each measure, then the parents provided their written responses to the items.

Demographic information on the children was obtained from hospital records and included age, gender, class affiliation, and diagnosis.

### 2.5. Statistical Analyses

The data were analyzed using random forest analysis [19,20], a versatile machine learning algorithm. There are two main reasons for using this approach. First, regression analysis is used to examine the influence of a small number of factors derived from a specific issue and does not provide a comprehensive comparison of the magnitude of influence of many factors. Second, regression analysis cannot simultaneously examine variables with high correlations due to the problem of multicollinearity. For these reasons, this study employed random forests, a part of machine learning that can comprehensively compare the influence of multiple factors and can simultaneously examine variables with relatively high correlations without being bound by multicollinearity. Random forest is a machine learning method that uses multiple trees to form a forest for identification. The trees are decision trees, and although individual decision trees do not have high discriminative performance, they can be used together to obtain high prediction performance. This is called ensemble learning in machine learning, and each decision tree corresponds to a weak discriminator in ensemble learning.

The general algorithm for random forests used in this study was as follows: (1) construct *n* bootstrap data sets from the original data; (2) generate *n* decision trees from the data set; (3) randomly select *m* features from *p* features; and (4) in the case of classification, the majority vote of *n* decision trees is used as the final prediction. In ensemble learning, the lower the correlation between models, the more accurate the predictions; therefore, only some of the features are used in the third step. The idea is that the degree of overlearning can be reduced by collecting decision trees overtrained in different directions and taking the average results.

As a procedural step, first, using a cut-off score of 75.3 points for the KINDL based on a previous study of Japanese children without disabilities [12], the children were classified into a high-QOL group (above average) and a low-QOL group (below average). These groups were used as the dependent variables. All PEM-CY items were used as the independent variables. Next, these variables were used to conduct a random forest regression, create a predictive model that classified the QOL into two categories, and extract the characteristic factors. The accuracy of the model was then further evaluated using cross-validation. Specifically, the data were divided into a 5-fold cross-validation, with the training and test data being classified at each fold and used as the training data to develop an algorithm to classify the QOL. R version 4.3.0 was used for data analysis.

Each PEM-CY item between the low- and high-QOL groups was also compared using the Mann–Whitney U test, for which IBM SPSS Statistics version 29.0.1 was used.

### 2.6. Ethical Considerations

The ethical considerations are the same in our previous research study [8]. All participants provided informed consent prior to their involvement in this study. This study adhered to the Declaration of Helsinki, and both the data collection methods and the study design received approval from the Ethics Committee of Chubu University (No: 20220095).

## 3. Results

### 3.1. Characteristics of the Children

Out of 137 parents of children who met the inclusion criteria and were invited to participate, 92 agreed to participate. The children’s demographic characteristics are presented in Table 1. The mean age was 7.6 (SD 1.6) years, and the majority (71%) of the children were male. Most of the children (61%) attended classes or schools where special support was provided, whereas 39% attended regular classes. The most frequent diagnoses included autism spectrum disorder (30%), developmental coordination disorder (24%), intellectual disorders (23%), specific learning disorders (15%), and attention deficit/hyperactivity disorder (ADHD) (8%). All participating parents were female with an average age of 40.7 (SD 4.0) years.

### 3.2. KINDL Scores

The KINDL scores of the participants are shown in Table 2. The mean total KINDL score was 69.3 (SD 10.6). The scores for each subscale were as follows: physical 82.7 (SD 13.2), emotional 77.5 (SD 14.2), self-esteem 57.7 (SD 20.4), family 65.7 (SD 13.4), friends 63.7 (SD 18.1), and school 68.7 (SD 17.3).

### 3.3. PEM-CY Scores

PEM-CY scores are shown in Table 3. Involvement and overall environmental support in the community setting were higher in the high-QOL group than in the low-QOL group, and the desire for change in the school setting was lower in the high-QOL group than in the low-QOL group.

### 3.4. PEM-CY Predictors of QOL

**Figure 1:**
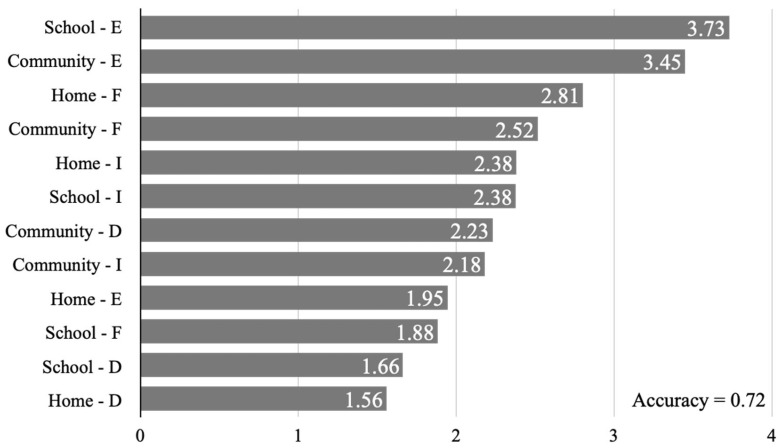
This graph shows the mean decrease in the Gini index for each PEM-CY item calculated using the random forest algorithm. The accuracy index, which indicates the accuracy of random forests, was 0.72 (72%). E: environmental support; F: frequency; I: involvement; D: desire for change.

The random forest results are illustrated in Figure 1. Comparisons of the mean decreases in the Gini index for each PEM-CY item indicated that the school environment (3.73) and the community environment (3.45) had the highest impact on QOL, followed by home frequency (2.81), community frequency (2.52), home involvement (2.38), and school involvement (2.38). In addition, the mean accuracy of this model was 0.72 (72%).

## 4. Discussion

### 4.1. Participation Predictors of QOL

Random forest algorithm analysis was employed in this study to analyze the relationships between participation in home, school, and community settings and QOL among Japanese children with NDDs. The results indicated that school environmental factors and community environmental factors that affected participation were the most important predictors of the children’s QOL. Previous studies found that environmental factors can significantly affect an individual’s ability to participate, particularly in children with a physical disability [5,6,17,21]. The results of this study confirm that this may also be true for children with NDDs; that is, enhancing environmental support could increase a child’s QOL. Environmental support was found to be context-specific to the school and community. Various environmental aspects were found to be supportive of or barriers to participation, such as physical (e.g., accessibility), social (e.g., peer support), attitudinal (e.g., perceptions toward disability), familial (e.g., family functioning), and institutional (e.g., policies) environments [21,22], all of which are included in the PEM-CY environmental items. Previous studies found that environment-focused approaches for children with cerebral palsy can be effective in improving their QOL [7,23,24]. Therefore, interventions to improve participation and QOL for children with NDDs should adopt an environment-focused approach. Our previous study suggests parents of children with NDDs should improve their children’s attitudinal environments [8]. The results of the current study indicate that support for participation is important to enhance the QOL and well-being of elementary school students with NDDs, and that environmental improvements are important to enable participation. This is underscored by the high mean accuracy of the random forest results.

Furthermore, when comparing the other Gini indexes for school and community, the frequency of participation was lower in the school than in the community. In school, the impact of the frequency of participation on QOL was minimal, as the Fundamental Law of Education [25] protects equal opportunity in education for all children. However, the results suggest that the frequency of community participation had an impact on the QOL of children with NDDs. Thus, it is necessary to support the frequency of community participation. However, involvement in activities was higher in school than in the community. This is where evaluation and comparison are necessary, as schools are required to carry out some of the same activities, namely educational activities. Therefore, the impact of school involvement on quality of life is considered high.

Home frequency was the next highest PEM-CY item that predicted QOL (home involvement was the fifth highest). Although environmental support at school and in the community is important for QOL, it is also important to enhance family participation. The PEM-CY assesses one private (home) and two public (school, community) settings. To improve the QOL of children with NDDs, it is important to focus participation interventions on both the public and private environments. Home participation scores have been found to be lower in children with autism spectrum disorder [15,26,27,28], ADHD [29], developmental coordination disorder [30], and specific learning disorders [31] compared to children without disabilities. Bowlby’s [32] concept of “a secure base” highlights the importance of home participation, primarily because enhancing home participation and making the home a secure base could lead to better participation in school and community environments.

The community had the highest rates of desire for change, with school and home having the lowest rates. This result suggests that support for community participation is needed. However, the desire for change is from the parent’s perspective and does not necessarily reflect the children’s desire for change. Therefore, further analysis that includes the children’s perspective is needed.

### 4.2. KINDL and PEM-CY Scores

The mean total KINDL score in the present study on Japanese children with NDDs was lower than the mean for Japanese children without disabilities in Furusho et al.’s [12] study. Comparatively, 73% had lower KINDL total scores. Several previous studies have found children with NDDs had a low QOL [33,34,35]; therefore, the results of our study are consistent with what has been observed in other countries.

PEM-CY scores indicated involvement and overall environmental support in the community setting was higher in the high-QOL group than in the low-QOL group, and desire for change in the school setting was lower in the high-QOL group than in the low-QOL group. A 2022 public opinion survey by Japan’s Cabinet Office [36] revealed that 88.5% of respondents believed that there was discrimination and prejudice against people with disabilities in Japan. Of these, 40.4% felt that discrimination and prejudice had not improved over the past five years [36]. Consequently, the stigma and exclusion faced by children with NDDs and their parents may contribute to challenges in community participation. However, there was no decrease in the desire for change in the community setting despite the low community involvement and overall environmental support. It was speculated that this may be due to guardian hermeneutical injustice, which is the injustice that occurs when significant aspects of an individual’s social experiences are obscured from collective understanding due to hermeneutical marginalization [37]. The parents of children with NDDs may believe they are not able to participate and that there is no environmental support; however, it is also possible that they limit their participation by believing that they would be a nuisance. Indeed, in our previous study, we analyzed the participation strategies of parents of children with NDDs using co-occurrence network analysis and the correspondence analysis [8]. Co-occurrence network analysis showed that “child”, “communicate”, “activity”, and “talk” were strongly related among the participation strategies in the home, school, and in community settings [8]. This indicates that advocating for child characteristics derived from NDDs is often used as a strategy to involve children in activities. Furthermore, the correspondence analysis results revealed that these strategies are specific to each setting [8]. There are specific injustices in each setting, which are not easy to address even with participation strategies. It is considered that this injustice, which is long-lasting and continues to befall children with NDDs and their parents, is reducing participation and QOL. In this situation, it is considered necessary to develop a participation-focused approach for children with NDDs and their parents that includes a social model, as a medical-model-based approach alone cannot solve this problem.

### 4.3. Limitations

This study sought to understand the restrictions on the QOL of Japanese children with NDDs by investigating participation factors. However, a limitation of this study was that all of the participants were from a single hospital. As such, the results may not be generalizable to other children with NDDs in Japan. Additionally, there is the limitation of sample bias with respect to age and diagnoses, which could have had an effect on the results. Therefore, in the future, it is necessary to conduct further validation studies at multiple facilities and control for age and diagnoses. In addition, children with NDDs were included in this study because most previous participation studies involved children with a physical disability [23,24]. Because NDDs are often a duplicate diagnosis, it makes it difficult to conduct a diagnosis-specific study. Nonetheless, it may be necessary in the future to study the target disorders separately. Lastly, a limitation of this study is that the results are only from a family perspective due to the nature of the questionnaire used.

## 5. Conclusions

This study aimed to clarify the relationship between QOL and participation for Japanese children with NDDs, thereby considering the need for a participation-focused approach. Therefore, this study used the KINDL and PEM-CY to survey the parents of Japanese children with NDDs. Data were analyzed using the random forest algorithm to identify the participation factors that could predict children’s QOL. The results indicated the importance of participation in school and community environments for the children’s QOL and the importance of participation at home. Interventions to improve participation and QOL for children with NDDs should adopt an environment-focused approach.

## Figures and Tables

**Table 1:** Characteristics of the children.

Children’s Demographics	*n* (%) or Mean (SD)
Gender, *n* (%)	
Male	65 (71)
Female	27 (29)
Age (years), mean (SD)	7.6 (1.6)
6 years, *n* (%)	33 (36)
7 years, *n* (%)	17 (18)
8 years, *n* (%)	13 (14)
9 years, *n* (%)	12 (13)
10 years, *n* (%)	11 (12)
11 years, *n* (%)	6 (7)
Type of class, *n* (%)	
Regular class	36 (39)
Special support services in resource room	12 (13)
Special support class	29 (32)
Special support school	15 (16)
Major diagnosis, *n* (%)	
Autism spectrum disorder	28 (30)
Attention deficit/hyperactivity disorder	7 (8)
Developmental coordination disorder	22 (24)
Specific learning disorders	14 (15)
Intellectual disorders	21 (23)

**Table 2:** KINDL total and subscale scores [8].

KINDL Scales	Mean (SD)
Total scale	69.3 (10.6)
Physical	82.7 (13.2)
Emotional	77.5 (14.2)
Self-esteem	57.7 (20.4)
Family	65.7 (13.4)
Friends	63.7 (18.1)
School	68.7 (17.3)

**Table 3:** PEM-CY scores.

Measure	Setting	Total (*n* = 92) Mean (SD)	Low-QOL Group (*n* = 67) Mean (SD)	High-QOL Group (*n* = 25) Mean (SD)	*p*-Value
Frequency (8-point scale)	Home School Community	5.58 (0.85) 3.97 (1.53) 1.97 (0.96)	5.50 (0.84) 3.91 (1.61) 1.92 (0.97)	5.70 (0.86) 4.14 (1.36) 2.10 (0.97)	0.26 0.37 0.45
Involvement (5-point scale)	Home School Community	3.90 (0.70) 3.51 (1.20) 3.18 (1.20)	3.87 (0.72) 3.33 (1.28) 3.12 (1.19)	3.99 (0.64) 4.00 (0.85) 3.34 (1.25)	0.27 0.72 0.02
Desire for change (percentage)	Home School Community	64 (28) 61 (36) 56 (31)	66 (28) 63 (34) 59 (30)	60 (30) 58 (43) 48 (33)	0.87 0.02 0.39
Overall environmental support (percentage)	Home School Community	80 (12) 84 (12) 81 (12)	79 (13) 83 (13) 79 (12)	83 (12) 88 (10) 85 (11)	0.48 0.18 0.04

QOL: quality of life. Mann–Whitney U test: *p* < 0.05.

## Data Availability

The data presented in this study are available on request from the corresponding author. The data are not publicly available due to privacy and ethical restrictions.
